# High-quality-draft genomic sequence of *Paenibacillus ferrarius* CY1^T^ with the potential to bioremediate Cd, Cr and Se contamination

**DOI:** 10.1186/s40793-017-0273-z

**Published:** 2017-10-10

**Authors:** Jingxin Li, Wei Guo, Manman Shi, Yajing Cao, Gejiao Wang

**Affiliations:** State Key Laboratory of Agricultural Microbiology, College of Life Science and Technology, Huazhong Agricultural University, Wuhan, 430070 People’s Republic of China

**Keywords:** *Paenibacillus ferrarius*, Genome sequence, Cadmium, Chromate-reducing bacterium, Selenite-reducing bacterium

## Abstract

*Paenibacillus ferrarius* CY1^T^ (= KCTC 33419^T^ = CCTCC AB2013369^T^) is a Gram-positive, aerobic, endospore-forming, motile and rod-shaped bacterium isolated from iron mineral soil. This bacterium reduces sulfate (SO_4_^2−^) to S^2−^, which reacts with Cd(II) to generate precipitated CdS. It also reduces the toxic chromate [Cr(VI)] and selenite [Se(VI)] to the less bioavailable chromite [Cr(III)] and selenium (Se^0^), respectively. Thus, strain CY1^T^ has the potential to bioremediate Cd, Cr and Se contamination, which is the main reason for the interest in sequencing its genome. Here we describe the features of strain CY1^T^, together with the draft genome sequence and its annotation. The 9,184,169 bp long genome exhibits a G + C content of 45.6%, 7909 protein-coding genes and 81 RNA genes. Nine putative Se(IV)-reducing genes, five putative Cr(VI) reductase and nine putative sulfate-reducing genes were identified in the genome.

## Introduction

The genus 10.1601/nm.5109 was established in 1993 with 10.1601/nm.5110 as the type species [1, 2]. The common characteristics of the 10.1601/nm.5109 members are aerobic, Gram-positive, rod-shaped and endospore-forming [3]. Some 10.1601/nm.5109 strains have the ability for plant growth promotion, biocontrol, manufacturing process and bioremediation, which making them very important in agricultural, industrial and medical applications [4]. A variety of industrial wastes including crude oil, diesel fuel, textile dyes, aliphatic and aromatic organic pollutants could be degraded by 10.1601/nm.5109 strains [5–11]. However, the bioremediation of heavy metal(loids) contamination by 10.1601/nm.5109 strains are rarely reported.

10.1601/nm.26197 CY1^T^ is a multi-metal(loids) resistant bacterium isolated from iron mineral soil in Hunan Province, China [12]. During cultivation, it could efficiently reduce sulfate (SO_4_^2−^) to S^2−^, which could precipitate with cadmium [Cd(II)] to generate CdS [13]. In addition, it also reduces the more toxic chromate [Cr(VI)] and selenite [Se(VI)] to the much less toxic chromite [Cr(III)] and selenium (Se^0^), respectively. Based on these interesting features, we propose that strain CY1^T^ represents a promising candidate for bioremediation of Cd, Cr and Se contamination. To gain insight into the molecular mechanisms involved in sulfate/chromate/selenite reduction and metal(loids) resistance, and to enhance its biotechnological applications, we analyze the high quality draft genome of this bacterium.

## Organism information

### Classification and features

**Fig. 1:**
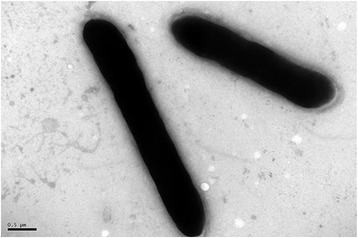
Scan electron microscope (SEM) image of *P. ferrarius* CY1^T^ cells. The bar scale represents 0.5 μm

**Fig. 2:**
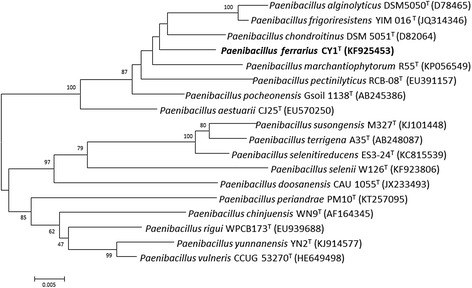
Phylogenetic tree depicting the relationship between *P. ferrarius* CY1^T^ and other members of the genus *Paenibacillus*. The phylogenetic tree was constructed based on the 16S rRNA gene sequences using neighbor-joining method (MEGA 6.0). The scale bar represents 0.01 nucleotide change per nucleotide position

10.1601/nm.26197 CY1^T^ is a Gram-positive, endospore-forming, motile and aerobic bacterium. The rod-shaped cells are 0.5–0.8 mm in width and 4.2–5.7 mm in length with peritrichous flagella (Fig. 1). Colonies are yellowish to creamy-white, smooth and circular on NA agar plate [12]. Growth occurs at temperature and pH range of 4–37 °C and pH 5.0–8.0, respectively [12]. Optimal growth occurs at 28 °C and pH 6.0–7.0 (Table 1). Strain CY1^T^ grows on NA/R2A/LB and TSA media, but cannot grow on MacConkey agar [12]. The phylogenetic relationship of 10.1601/nm.26197 CY1^T^ with other members within the genus 10.1601/nm.5109 is shown in a 16S rRNA based neighbor-joining tree, and strain CY1^T^ is closely related to 10.1601/nm.28428 R55 ^T^ (KP056549) (Fig. 2).

**Table 1:** Classification and general features of *Paenibacillus ferrarius* CY1^T^

MIGS ID	Property	Term	Evidence code^a^
	Classification	Domain *Bacteria*	TAS [39]
		Phylum *Firmicutes*	TAS [40–42]
		Class *Bacilli*	TAS [43, 44]
		Order *Bacillales*	TAS [45, 46]
		Family *Paenibacillaceae*	TAS [44]
		Genus *Paenibacillus* Species *Paenibacillus ferrarius*	TAS [1, 47–50] IDA
		Strain CY1^T^	IDA
	Gram stain	Positive	IDA
	Cell shape	Rod	IDA
	Motility	Motile	IDA
	Sporulation	Endospore	IDA
	Temperature range	4–37 °C	IDA
	Optimum temperature	28 °C	IDA
	pH range; Optimum	5–8; 6–7	IDA
	Carbon source	Rhamnose, glycogen, sucrose N-acetylglucosamine, maltose, mannitol, D-glucose, salicin, melibiose, D-sorbitol, L-arabinose, mannose, D-xylose, ammonium nitrate and L-proline	IDA
MIGS-6	Habitat	Soil	IDA
MIGS-6.3	Salinity	0–1.5% NaCl (*w*/*v*)	IDA
MIGS-22	Oxygen requirement	Aerobic	IDA
MIGS-15	Biotic relationship	Free-living	IDA
MIGS-14	Pathogenicity	Non-pathogen	NAS
MIGS-4	Geographic location	Zhangjiajie city, Hunan province, China	IDA
MIGS-5	Sample collection	2013	IDA
MIGS-4.1	Latitude	N29°35’	IDA
MIGS-4.2	Longitude	E110°54’	IDA
MIGS-4.4	Altitude	860 m	IDA

^a^Evidence codes - *IDA* inferred from direct assay, *TAS* traceable author statement (i.e., a direct report exists in the literature), *NAS* non-traceable author statement (i.e., not directly observed for the living, isolated sample, but based on a generally accepted property for the species, or anecdotal evidence). These evidence codes are from the Gene Ontology project [51]

Physiological and biochemical analyses were performed using the API 20NE test (bioMérieux, France), ID 32GN text (bioMérieux, France) and traditional classification methods. Strain CY1^T^ is positive for oxidase and catalase activities, hydrolysis of Tween 80 and aesculin and production of NH_3_ and H_2_S, but is negative for nitrate reduction, citrate utilization, egg yolk reaction, production of indole, and hydrolysis of starch, gelatin, casein, urea, L-tyrosine, arginine, Tween 20, DNA and CM-cellulose [12]. The carbon sources, which can be used by strain CY1^T^, are shown in Table 1.

**Fig. 3:**
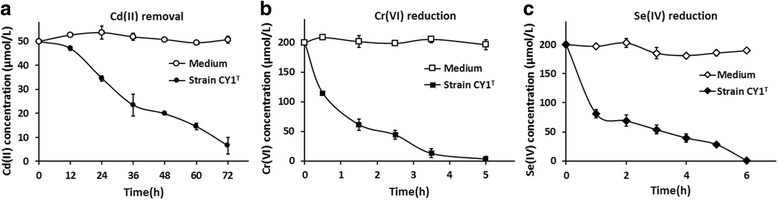
Cd(II) removal (**a**), and Cr(VI) (**b**) and Se(IV) (**c**) reduction by *P. ferrarius* CY1^T^. Strain CY1^T^ was incubated in LB [for Cd(II) removal] or NA medium [for Cr(VI) and Se(IV) reduction] until OD_600_ reach 0.6-0.7, and then amended with CdCl_2_ (50 μmol/L),K_2_CrO_4_ (200 μmol/L) and Na_2_SeO_3_ (200 μmol/L), respectively. At designed times, culture samples were taken for measuring the residual concentration of Cd(II), Cr(VI) and Se(IV). Data are shown as the mean of three replicates, with the error bars represents ± SD

The resistance levels of 10.1601/nm.26197 CY1^T^ for multi-metal(loids) were tested with the minimal inhibition concentration on NA agar plates using Na_3_AsO_3_, K_2_Sb_2_(C_4_H_2_O_6_)_2_, Na_2_SeO_3_, K_2_CrO_4_, CdCl_2_, PbCl_2_, CuCl_2_ and MnCl_2_. The results showed that the MICs for As(III), Sb(III), Se(IV), Cr(VI), Cd(II), Pb(II), Cu(II) and Mn(II) are 2, 1, 8, 4, 0.08, 1, 0.5 and 100 mmol/L, respectively. In addition, the abilities of strain CY1^T^ for Cd(II) removal, and Cr(VI) and Se(IV) reduction were tested. Strain CY1^T^ was incubated in LB medium for Cd(II) removal and in NA medium for Cr(VI) and Se(IV) reduction, since NA medium can absorb some of the Cd(II). When OD_600_ reach 0.6-0.7, CdCl_2_ (50 μmol/L), K_2_CrO_4_ (200 μmol/L) and Na_2_SeO_3_ (200 μmol/L) were each added to the culture. At designated times, culture samples were taken for measuring the residual concentrations of Cd(II), Cr(VI) and Se(IV). The concentration of Cd(II) was measured by the atomic absorption spectrometry [14]. The concentration of Cr(VI) was measured by the UV spectrophotometer (DU800, Beckman, CA, USA) with the colorimetric diphenylcarbazide method [15], and the concentration of Se(IV) was tested by HPLC-HG-AFS (Beijing Titan Instruments Co., Ltd., China) [16]. The results showed that strain CY1^T^ could remove nearly 50 μmol/L Cd(II) in 72 h (Fig. 3a) and reduce 200 μmol/L Cr(VI) and Se(IV) in 5 h and 6 h, respectively (Fig. 3b, c). The removed Cd(II) is presented as pellets that is most probably by the reaction of Cd(II) with H_2_S to produce precipitated CdS.

## Genome sequencing information

### Genome project history

Strain CY1^T^ was selected for genome sequencing on the basis of its ability for Cd(II) removal, Cr(VI) and Se(IV) reduction, these characters made strain CY1^T^ with great value for genetic study and for bioremediation of Cd, Cr and Se contamination. The draft genome sequence is deposited at DDBJ/EMBL/GenBank under the accession number MBTG00000000. The final genome consists of 73 scaffolds with 289.77 × coverage. A summary of the project information is shown in Table 2.

**Table 2:** Project information

MIGS ID	Property	Term
MIGS-31	Finishing quality	High-quality draft
MIGS-28	Libraries used	Illumina Paired-End library (300 bp insert size)
MIGS-29	Sequencing platforms	Illumina Miseq 2000
MIGS-31.2	Fold coverage	289.77 ×
MIGS-30	Assemblers	SOAPdenovo v2.04
MIGS-32	Gene calling method	GeneMarkS^+^
	Locus TAG	BC351
	Genbank ID	MBTG00000000
	Genbank Date of Release	Mar 16, 2017
	Bioproject	PRJNA331076
MIGS-13	Source material identifier	Strain KCTC 33419^T^ (CCTCC AB2013369^T^)
	Project relevance	Bioremediation

### Growth conditions and genomic DNA preparation

Overnight cultures of strain CY1^T^ was inoculated into 50 mL of NA medium at 28 °C with 120 rpm shaking. After incubation for 36 h, the bacterial cells were harvested through centrifugation (13,400×g for 5 min at 4 °C). Genomic DNA was extracted using the QiAamp kit (Qiagen, Germany). The quality and quantity of the DNA were determined by a spectrophotometer (NanoDrop 2000, Thermo). Then, 10 μg of DNA was sent to Bio-broad Technology Co., Ltd., Wuhan, China for sequencing.

### Genome sequencing and assembly

Genome sequencing and assembly were performed by Bio-broad Technology Co., Ltd., Wuhan, China, and all original sequence data can be found at the NCBI Sequence Read Archive. An Illumina standard shotgun library was constructed and sequenced using an Illumina Hiseq2000 platform with pair-end sequencing strategy (300 bp insert size) [17]. The following quality control steps were performed for removing low quality reads: 1) removed the adapter sequences of reads; 2) trimmed the ambiguous bases (N) in 5′ end and the reads with a quality score lower than 20; and 3) filtered the reads which contain N more than 10% or have the length less than 50 bp (without adapters and N in 5′ end). The assembly of CY1^T^ genome is based on 20,189,278 quality reads totaling 3,000,798,615 bp, which provides a coverage of 289.77×. Subsequently, the reads were assembled into 75 contigs (> 200 bp) using SOAPdenovo v2.04 [18], and the gaps between the contigs were closed by GapCloser v1.12 [19].

### Genome annotation

The draft genome of strain CY1^T^ was annotated through the RAST server version 2.0 and the NCBI Prokaryotic Genome Annotation Pipeline. Genes were identified using the gene caller GeneMarkS^+^ with the similarity-based gene detection approach [20]. Pseudogenes were also predicted using the NCBI PGAP. Internal gene clustering was performed by OrthoMCL using Match cutoff of 50% and E-value Exponent cutoff of 1-e5 [21, 22]. The COGs functional categories were assigned by WebMGA server [23] with E-value cutoff of 1-e10. The translations of the predicted CDSs were used to search against the Pfam protein family database [24] and the KEGG database [25]. The transmembrane helices and signal peptides were predicted by TMHMM v. 2.0 [26] and SignalP 4.1 [27], respectively.

## Genome properties

The whole genome of strain CY1^T^ reveals a genome size of 9,184,169 bp and a G + C content of 45.6% (Table 3). The genome contains 8260 coding sequences, 19 rRNA, 58 tRNA, and 4 ncRNA. Among 7909 protein-coding genes, 4231 were assigned as putative function, while the other 3678 were designated as hypothetical proteins. In addition, 6632 genes were categorized into COGs functional groups. Information about the genome statistics is shown in Table 3 and the classification of genes into COGs functional categories is summarized in Table 4.

**Table 3:** Genome statistics

Attribute	Value	% of total^a^
Genome size (bp)	9,184,169	100.00
DNA coding (bp)	7,828,640	85.24
DNA G + C (bp)	4,205,829	45.79
DNA scaffolds	73	100.00
Contigs	75	100.00
Total genes^b^	8260	
RNA genes	81	
Pseudo genes	209	
Protein-coding genes	7909	100.00
Genes in internal clusters	648	8.19
Genes with function prediction	4231	53.50
Genes assigned to COGs	6632	83.85
Genes with Pfam domains	6363	80.45
Genes with signal peptides	765	9.67
Genes with transmembrane helices	2251	28.46
CRISPR repeats	24	0.30

^a^The total is based on either the size of the genome in base pairs or the total number of protein coding genes in the annotated genome

^b^Also includes 209 pseudogenes, 58 tRNA genes, 19 rRNAs and 4 ncRNA

**Table 4:** Number of genes associated with general COG functional categories

Code	Value	% of total^a^	Description
J	199	2.52	Translation, ribosomal structure and biogenesis
A	0	0.00	RNA processing and modification
K	732	9.26	Transcription
L	213	2.69	Replication, recombination and repair
B	1	0.01	Chromatin structure and dynamics
D	55	0.70	Cell cycle control, cell division, chromosome partitioning
Y	0	0.00	Nuclear structure
V	128	1.62	Defense mechanisms
T	694	8.77	Signal transduction mechanisms
M	328	4.15	Cell wall/membrane/envelope biogenesis
N	107	1.35	Cell motility
Z	11	0.14	Cytoskeleton
U	63	0.80	Intracellular trafficking, secretion, and vesicular transport
O	146	1.85	Posttranslational modification, protein turnover, chaperones
C	268	3.39	Energy production and conversion
G	1023	12.93	Carbohydrate transport and metabolism
E	432	5.46	Amino acid transport and metabolism
F	121	1.53	Nucleotide transport and metabolism
H	194	2.45	Coenzyme transport and metabolism
I	149	1.88	Lipid transport and metabolism
P	361	4.56	Inorganic ion transport and metabolism
Q	134	1.69	Secondary metabolites biosynthesis, transport and catabolism
R	777	9.82	General function prediction only
S	496	6.27	Function unknown
**–**	1277	16.15	Not in COGs

^a^The total is based on the total number of protein coding genes in the annotated genome

## Insights from the genome sequence

**Fig. 4:**
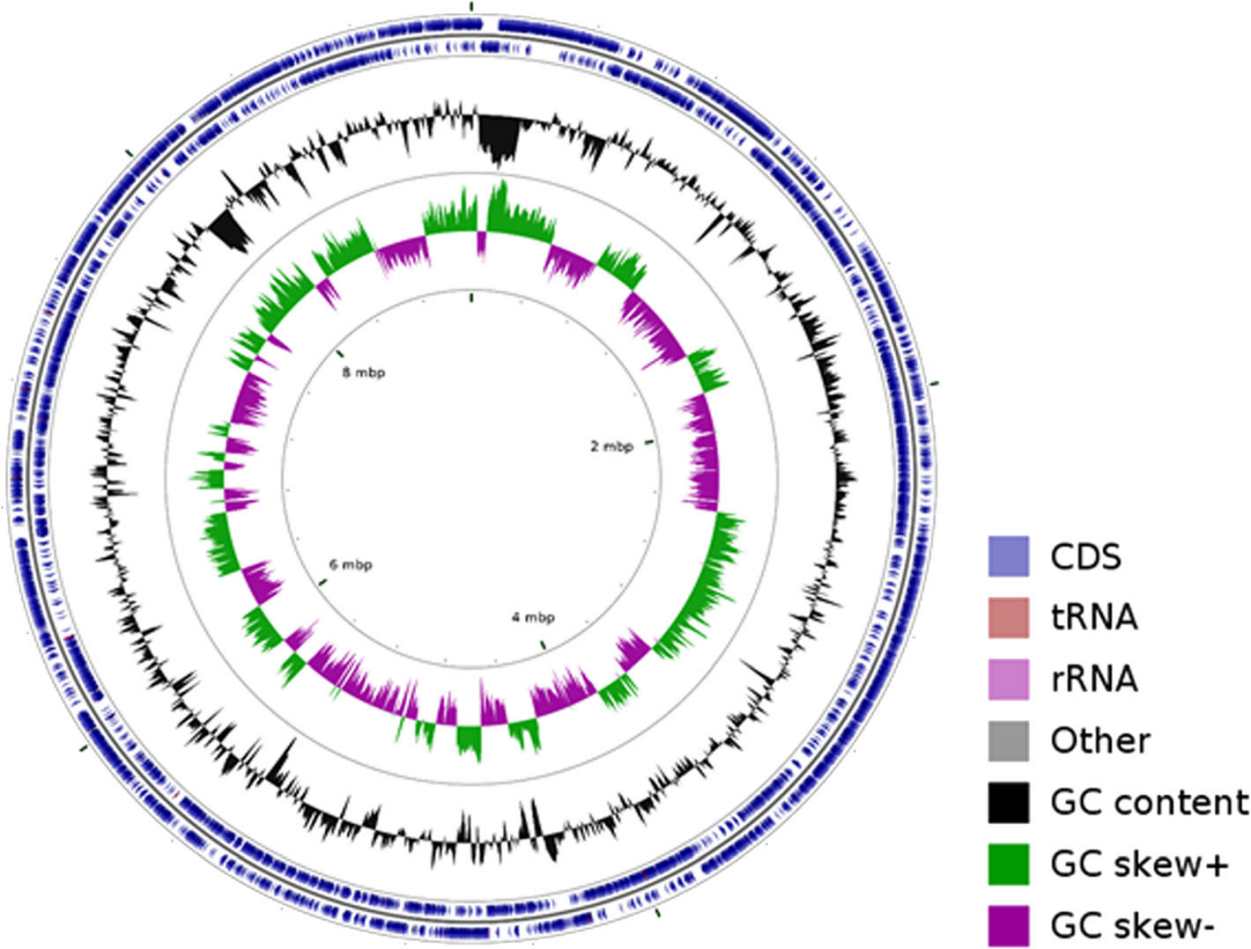
A graphical circular map of strain *P. ferrarius* CY1^T^. From outside to center, rings 1 and 2 denotes the predicted coding sequences on forward/reverse strand with each gene colored by its assigned COG category; ring 3 shows G + C % content plot and ring 4 shows GC skew

10.1601/nm.26197 CY1^T^ is a multi-metal(loids) resistant bacterium with the capability of SO_4_^2−^, Cr(VI) and Se(IV) reduction, suggesting that it has developed a number of evolutionary strategies to adapt to heavy metal (or metalloids) contaminated environments. To identify pathways and enzymes involved in SO_4_^2−^, Cr(VI) and Se(IV) reduction, high quality draft genome sequence of strain CY1^T^ was generated. The map of the 10.1601/nm.26197 CY1^T^ genome is shown in Fig. 4.

KEGG analysis showed that strain CY1^T^ contains a complete SO_4_^2−^ reduction pathway, which is consistent with the phenotype of H_2_S production. The genes responsible for SO_4_^2−^ reduction include sulfate ABC transporter CysPWA, sulfate adenylyltransferase CysD, adenylylsulfate kinase CysC, adenylylsulfate reductase CysH and sulfite reductase CysJI (Table 5). The S^2−^ generated from SO_4_^2−^ reduction could react with Cd(II) to form the participated CdS [13], which may contribute to the Cd(II) removal. For Cr(VI) reduction, five NADPH-dependent FMN reductase which have the same conserved domain as the Cr(VI) reductases ChrR (from 10.1601/nm.2674) and YieF (from 10.1601/nm.3093) [28], were identified in the genome of strain CY1^T^ (Table 5). It has been reported that thioredoxin reductase ThxR and NADH:flavin oxidoreductase could reduce Se(IV) in 10.1601/nm.22134 and 10.1601/nm.13570, respectively [29–31]. According to the NCBI and RAST annotation, seven thioredoxin reductases and two NADH-dependent flavin oxidoreductases were found in the genome of strain CY1^T^ (Table 5), and some of these proteins may responsible for Se(IV) reduction in strain CY1^T^.

**Table 5:** Putative proteins involved in selenite, chromate and sulfate reduction

Metal(loids)	Putative function	Locus_tag of the predicted protein
Selenite	Thioredoxin reductase	BC351_25440
	Thioredoxin reductase	BC351_17745
	Thioredoxin reductase	BC351_21345
	Thioredoxin reductase	BC351_06135
	Thioredoxin reductase	BC351_33000
	Thioredoxin reductase	BC351_13625
	Thioredoxin-disulfide reductase	BC351_19150
	NADH-dependent flavin oxidoreductase	BC351_22155
	NADH-dependent flavin oxidoreductase	BC351_12795
Chromate	NADPH-dependent FMN reductase	BC351_21415
	NADPH-dependent FMN reductase	BC351_05445
	NADPH-dependent FMN reductase	BC351_40245
	NADPH-dependent FMN reductase	BC351_15505
	NADPH-dependent FMN reductase	BC351_15285
Sulfate	Sulfate adenylyltransferase small subunit CysD	BC351_30725
	Adenylyl-sulfate kinase CysC	BC351_31925
	Adenylyl-sulfate kinase CysC	BC351_32075
	Phosphoadenosine phosphosulfate reductase CysH	BC351_36025
	Sulfate ABC transporter substrate-binding protein CysP	BC351_12315
	Sulfate ABC transporter CysA	BC351_12325
	Sulfate ABC transporter permease subunit CysW	BC351_12330
	Sulfite reductase alpha component	BC351_31155
	Sulfite reductase beta subunit	BC351_31160

Strain CY1^T^ could tolerant multi-metal(loids), such as As(III), Sb(III), Cr(VI), Cd(II), Pb(II), Cu(II) and Mn(II). Expectably, various metal resistant genes were identified in its genome (Table 6). Several transporters were found to responsible for the efflux of these metal(loids). In addition, the transcriptional regulator ArsR and arsenite reductase ArsC were also found to be involved in the As(III)/Sb(III) resistance (Table 6) [32–34]. Recently, it has been reported that an oxidoreductase AnoA, which belongs to the short-chain dehydrogenase/reductase family, and catalase KatA, which is responsible for H_2_O_2_ degradation, are all involved in bacterial Sb(III) oxidation/resistance in 10.1601/nm.1311 GW4 [35–38]. One AnoA homologue oxidoreductase gene and five catalase genes were identified in the genome of strain CY1^T^ (Table 6), which may associate with Sb(III) oxidation/resistance.

**Table 6:** Putative proteins involved in metal(loid) resistance

Heavy metal	Putative function	Locus_tag of the predicted protein
Arsenic	Arsenic transporter	BC351_03410
	Arsenical efflux pump membrane protein ArsB	BC351_32265
	Arsenic ABC transporter ATPase	BC351_35545
	ArsR family transcriptional regulator	BC351_32260
	ArsR family transcriptional regulator	BC351_02635
	Arsenate reductase ArsC	BC351_15540
Antimony	Oxidoreductase (putative AnoA)	BC351_17295
	Catalase	BC351_40130
	Catalase	BC351_06195
	Catalase	BC351_15905
	Catalase	BC351_07965
	Catalase	BC351_29865
Chromate	ChrA protein	BC351_26450
	Chromate transporter	BC351_15935
	Chromate transporter	BC351_29720
	Chromate transporter	BC351_29725
Cadmium, lead and zinc	Cobalt-zinc-cadmium resistance protein	BC351_15845
	Cobalt-zinc-cadmium efflux system protein	BC351_17600
	Cation diffusion facilitator family transporter	BC351_20420
	Cation diffusion facilitator family transporter	BC351_03295
	RND family efflux transporter	BC351_25240
	RND family efflix transporter/ MFP transporter	BC351_17480
	RND family efflux transporter, MFP subunit	BC351_10185
	Efflux transporter periplasmic adaptor subunit	BC351_04820
	Efflux transporter periplasmic adaptor subunit	BC351_25355
	Cd^2+^/Zn^2+^-exporting ATPase \cadmium transporter	BC351_28470
	HlyD family secretion protein	BC351_33510
	HlyD family secretion protein\ MFP transporter	BC351_35605
	Multidrug efflux pump subunit AcrA	BC351_02380
	Efflux transporter periplasmic adaptor subunit	BC351_37435
	Cation transporter	BC351_08750
	Zinc transporter ZitB	BC351_12865
	Cadmium transporter	BC351_35590
	Cadmium-translocating P-type ATPase	BC351_14640
Copper	Bcr/CflA family drug resistance efflux transporter	BC351_19565
	Multidrug resistance transporter, Bcr/CflA family	BC351_07275
	Copper transport protein	BC351_15720
	Copper-translocating P-type ATPase	BC351_26145
	Copper-translocating P-type ATPase	BC351_38485
	Copper-transporting P-type ATPase CopZ	BC351_38480
Manganese	Manganese transport protein MntH	BC351_25600
	Manganese transport protein MntH	BC351_14100

## Conclusions

The genome of 10.1601/nm.26197 CY1^T^ harbors various genes responsible for sulfate transport and reduction, chromate and selenite reduction and resistance of multi-metal(loids), which is consistent with its phenotypes. To date, the utilization of 10.1601/nm.5109 species in immobilization of heavy-metals (or metalloids) is still limited and the genes and enzymes involves in Cr(VI) and Se(IV) reduction were poorly understood in 10.1601/nm.5109 members. The genomic sequence of strain CY1^T^ enriches the genome information of 10.1601/nm.5109 strains. More importantly, the genome information provides basis for understanding molecular mechanisms of microbial redox transformations of metal(loids).
